# Induction of Heme Oxygenase-1 Modifies the Systemic Immunity and Reduces Atherosclerotic Lesion Development in ApoE Deficient Mice

**DOI:** 10.3389/fphar.2022.809469

**Published:** 2022-02-24

**Authors:** Leyi Yao, Yali Hao, Guanmei Wen, Qingzhong Xiao, Penglong Wu, Jinheng Wang, Jinbao Liu

**Affiliations:** ^1^ Guangzhou Municipal and Guangdong Provincial Key Laboratory of Protein Modification and Degradation, School of Basic Medical Sciences, Affiliated Cancer Hospital and Institute of Guangzhou Medical University, Guangzhou Medical University, Guangzhou, China; ^2^ Institute of Digestive Disease of Guangzhou Medical University, Qingyuan People’s Hospital, The Sixth Affiliated Hospital of Guangzhou Medical University, Qingyuan, China; ^3^ Guangdong Key Laboratory of Vascular Diseases, State Key Laboratory of Respiratory Disease, Guangzhou Institute of Cardiovascular Disease, The Second Affiliated Hospital, Guangzhou Medical University, Guangzhou, China; ^4^ Clinical Pharmacology, Barts and The London School of Medicine and Dentistry, William Harvey Research Institute, Queen Mary University of London, London, United Kingdom; ^5^ Hospital of Xiamen University, School of Medicine, Xiamen University, Xiamen, China

**Keywords:** heme oxygenase-1, atherosclerosis, systemic immunity, hemin, SNPP

## Abstract

Heme oxygenase-1 (HO-1) has been reported to protect against oxidation and inflammation in atherosclerosis. It remains unclear how the immune system participates in the cytoprotective function of HO-1 in the context of atherosclerosis. In this study, we attempted to investigate the potential effect of a HO-1 inducer, hemin, and a HO-1 inhibitor, Tin-protoporphyrin IX (SnPP), on the progression of atherosclerosis in ApoE deficient mice. Using mass cytometry, 15 immune cell populations and 29 T cell sub-clusters in spleen and peripheral blood were thoroughly analyzed after hemin or SnPP treatment. SnPP elevated risk factors of atherosclerosis, whereas hemin reduced them. In-depth analysis showed that hemin significantly modified the immune system in both spleen and peripheral blood. Hemin increased dendritic (DC) and myeloid-derived suppressor cells (MDSCs), but decreased natural killer (NK) cells. An opposite effect was observed with SnPP treatment in terms of NK cells. NK cells and MDSCs were positively and negatively correlated with total cholesterol and low-density lipoprotein, respectively. Moreover, the T cell profiles were significantly reshaped by hemin, whereas only minor changes were observed with SnPP. Several hemin-modulated T cell clusters associated with atherosclerosis were also identified. In summary, we have unraveled an important regulatory role for HO-1 pathway in immune cell regulation and atherosclerosis. Our finding suggests that modulating HO-1 signaling represents a potential therapeutic strategy against atherosclerosis.

## Introduction

Heme oxygenase-1 (HO-1) is a well-characterized inducible isoform of the rate-limiting monoxide, ferrous iron, and biliverdin. While some immune cells including iron-recycling macrophages in the spleen and liver and tolerogenic immune cells constitutively express HO-1, most other tissues and cells express little or no HO-1 under homeostatic conditions (Campbell et al., 2021). In response to pro-oxidant stimuli, HO-1 is highly upregulated in most cell types, protecting them from oxidative damage. As a result of its antioxidant properties, HO-1 has cytoprotective effects in various pathological conditions such as atherosclerosis, diabetes, and occlusive vascular disease (Durante, 2011).

Atherosclerosis is a chronic inflammatory disease associated with multifactorial mechanisms, which are primarily driven by oxidative stress and inflammation (Agudiez et al., 2020; Dinh et al., 2017; Le, 2004; Wang et al., 2014). Apolipoprotein E knockout (ApoE^-/-^) mice and low-density lipoprotein (LDL) receptor deficient (LDLr^−/-^) mice are widely used as murine models for atherosclerosis (Mukhopadhyay, 2013). Both *in vivo* and *in vitro* evidence have demonstrated the protective properties of HO-1 against atherosclerosis. In a co-culture system of human aortic endothelial cells and smooth muscle cells, pretreatment with HO-1 inducer hemin chloride suppressed monocyte chemotaxis induced by oxidized low-density lipoprotein (Ishikawa et al., 1997). By inhibiting MCP-1 production (Shokawa et al., 2006), foam cell formation, and pro-inflammatory cytokine production or by upregulating anti-inflammatory mediators, HO-1 may act as an anti-atherogenic molecule (Ma et al., 2007). In an animal model, HO-1 deficiency resulted in more advanced atherosclerotic lesions in ApoE^-/-^ mice (Wu et al., 2006). The inhibition of HO-1 by pharmacological agents like Tin-protoporphyrin IX (SnPP), exacerbates atherosclerosis in LDLr^−/-^ mice (Ishikawa et al., 2001b) or heritable hyperlipidemic rabbits (Ishikawa et al., 2001a). Overexpression of HO-1 *via* adenovirus-mediated gene transfer in vascular cells attenuates the development of atherosclerosis in ApoE^-/-^ mice (Juan et al., 2001). The level of HO-1 expression is positively associated with the risk and severity of atherosclerosis. A higher level of HO-1 in monocyte-derived macrophages was related to the unstable phenotype of atherosclerotic plaques featured with higher content of macrophage, thin fibrous caps, and thrombosis (Fiorelli et al., 2019). HO-1’s anti-atherogenic properties may also be mediated through free radicals scavenging, fatty acid oxidation inhibition, lipid composition modification, or influence on the nitric oxide pathway (Durante, 2011; Bellner et al., 2020). In addition to its antioxidant effects, HO-1 has been widely reported to have anti-inflammatory effects in atherosclerosis. HO-1 is upregulated during inflammation and subsequently suppresses vascular inflammation by affecting a variety of immune cells, including macrophages, dendritic cells, T cells, and mast cells (Ryter, 2021). HO-1 has been well documented for its cardioprotective properties, but its effects on systemic immunity are not well understood. As a chronic inflammatory disease, atherosclerosis is closely associated with immune modulation.

Mass cytometry, an emerging technology, has been widely applied in recent years to analyze immune systems at the single cell level (Bandura et al., 2009; Bodenmiller et al., 2012). Traditional flow cytometry is restricted by a limited number of detection channels (generally <15) and cumbersome compensation caused by spectral overlap, whereas mass cytometry can simultaneously measure more than 40 parameters in millions of individual cells (Bendall et al., 2011). The expansion of channels enables us to quantify more surface and intracellular proteins in single cell and thus facilitate in-depth analyses of cellular heterogeneity and comprehensive dissections of immune systems (McGuire et al., 2020). Mass cytometry has already been used to expand our understanding of complex processes in cellular development (Bendall et al., 2014), tumor immunology (Wang et al., 2020a; Wang et al., 2020b), and immune modulation in cardiovascular disease (Cole et al., 2018).

ApoE^−/−^ mice exhibit higher plasma total cholesterol level than LDLr^-/-^ mice on a chow diet, and develop severe atherosclerotic lesions and proinflammatory changes as soon as a few weeks, making them good candidates for studying inflammation of atherosclerosis (Lo Sasso et al., 2016). In this study, we used the well-characterized HO-1 inducer hemin and inhibitor SnPP to examine the protective role of HO-1 in the ApoE^−/−^ atherosclerosis mouse model. Following hemin or SnPP treatment, mass cytometry was utilized to simultaneously assess the expression of 26 immune cell markers on spleen and peripheral blood immune cells at the single cell level. Additionally, high-dimensional analysis was used to uncover the effects of HO-1 on systemic immune regulation in the context of atherosclerosis.

## Methods and Materials

### Animals

8-week-old male ApoE^-/-^ mice (C57BL/6.129P2-APOE/J) were purchased from Beijing Vital River Laboratory Animal Technology. These mice were housed and treated following the protocols approved by the Institutional Committee for the Use and Care of Laboratory Animals of Guangzhou Medical University (2017-014). All animals were fed on a western diet supplemented with 21% fat (wt/wt) and 0.15% cholesterol (wt/wt) at 8 weeks of age and this diet was continued for 10 weeks. ApoE^-/-^ mice were simultaneously intraperitoneally injected with HO-1 inducer hemin (Hemin group, 51280, Sigma, 5 mg/kg/d, *n* = 6) or HO-1 inhibitor SnPP (SnPP group, B6432, APExBIO, 10 mg/kg/d, *n* = 10) or vehicle (Control group, *n* = 8) every other day for 10 weeks, during which time the body weight and the daily intake of food and water were monitored at weekly interval. The mice were euthanized with an overdose of pentobarbital sodium (200 mg/kg, Sigma, St. Louis, MO, United States) for blood and tissue collection.

### Biochemical Assays

Peripheral blood was collected into a heparin-coated tube by cardiac puncture at the end (10 weeks) of the study for biochemical assay. All metabolic parameters, including glucose, lipid or lipoprotein profile, and hepatic enzymes in plasma, were determined by following the instructions of the commercial kits (Kehua Bio-Engineering, Shanghai, China).

### Histological and Morphometric Analyses

The hearts perfused with 5 ml phosphate-buffered saline were dissected and snap-frozen in liquid nitrogen followed by OCT embedment. The entire aorta between the heart and iliac bifurcation was carefully dissected. Aorta segments, including the ascending aorta, aorta arch, descending thoracic and abdominal aortas, were separated and fixed in formaldehyde solution at room temperature (RT) for 24 h. Under a stereomicroscope (SXZ 16, OLYMPUS, JAPAN), the brachiocephalic trunk was dissected from the aorta arch. The rest of the aorta segments were dissected free of fat tissue and opened longitudinally. To prepare the stock solution, 0.25 g of oil red O (ORO, O026, Sigma, St. Louis, MO, United States) was dissolved in 100 ml of distilled water. The working solution is prepared by adding four parts of distilled water to six parts of the stock solution. After rinsing with 60% isopropyl alcohol for 5 min, the fixed arterial tree was incubated in a working solution at room temperature for 30 min. The sample was then rinsed in 60% IPA, followed by distilled water. After staining with ORO, the aorta was pinned onto a black wax plate and photographed under a Stereomicroscope at standardized magnification and illumination. The aorta lesion area was quantified as the percentage of ORO red positive area over the total aorta area using ImageJ software. The heart and brachiocephalic trunk were serially sectioned into 5 μm cryosections. The first section of the aortic sinus was harvested when all three aortic valves became visible in the lumen of the aorta. The cryosections were subjected to hematoxylin and eosin (H&E, KGA223, KeyGEN, Jiangshu, China) staining for morphological analysis, ORO staining for lipid load measurement, and Masson’s Trichrome (DC0033, LEAGENE, Beijing, China) staining for collagen detection, respectively. Microscopic atherosclerotic lesions in the aortic sinus were standardized as the ratio of intima area to the media area. All images were captured by a digital pathology slide scanner (Leica, Aperio CS2, Germany).

### Peripheral Blood and Spleen Cell Preparation

After injection of pentobarbital sodium, peripheral blood was collected from the heart. The spleen of mice was removed after sacrifice, and the spleen cell suspension was separated by gently crushing the spleen. Fix I buffer (Fluidigm) was used for 10 min at room temperature to fix cells from peripheral blood or spleen. After washing with cold phosphate buffered solution, erythrocytes were removed from these samples using red blood cell lysis buffer. Cells were then resuspended in cell staining buffer supplemented with 10% dimethyl sulphoxide and stored at −80°C until cell staining was performed.

### Immunostaining and Mass Cytometry

To eliminate sample-specific staining variation, 0.5 × 10^6^ peripheral blood or spleen cells from each mouse were barcoded separately using the Cell-ID 20-Plex Pd Barcoding Kit (Fluidigm) containing premade combinations of six different palladium isotopes for 30 min at room temperature. After washing twice with cell staining buffer, samples were pooled together. To reduce nonspecific antibody binding, samples were incubated for 10 min at RT with anti-CD16/32 antibody (FcR III/II, Biolegend, CA, United States). A cocktail of 26 metal isotope-conjugated antibodies (Supplementary Table S1) was incubated with these cells for 30 min at room temperature after washing twice with staining buffer. Cell-ID Intercalator-Ir (Fluidigm) was used to stain the cell nucleus. After washing with cell staining buffer and ddH2O, cells were resuspended in ultrapure water supplemented with 10% EQ Four Element Calibration Beads (Fluidigm). The sample was filtered through a 40-μm cell strainer and analyzed using Helios mass cytometer (Fluidigm).

### Data Processing and Analysis

Flow cytometry standard (FCS) files were generated by mass cytometry, and data were normalized, randomized, and debarcoded using the CyTOF 6.7 software (Fluidigm). These files were uploaded to cytobank. cn for cytometry-based single cell analysis. Cell debris and doublets were excluded and the single cell data were used for high-dimensional analysis with viSNE algorithm. We directly gated clear cell populations based on marker expression on cells in the viSNE map and on phenotypes of immune cell populations or T cell subsets. Also, two dimensional plots were used to distinguish the other cell populations from the rest of the cells in the viSNE map.

### Statistical Analysis

One-way ANOVA followed by Tukey’s multiple comparison test was used for comparing multiple groups. Person’s correlation coefficient was used for correlation analyses. |R|>0.4 with *p* < 0.05 was regarded as statistical correlation. Data presented as the mean ± SD. *p* < 0.05 was regarded as statistically significant.

## Results

### The HO-1 Inducer Hemin Improves Atherogenesis and its Inhibitor SnPP Exacerbates Atherogenesis

**FIGURE 1:**
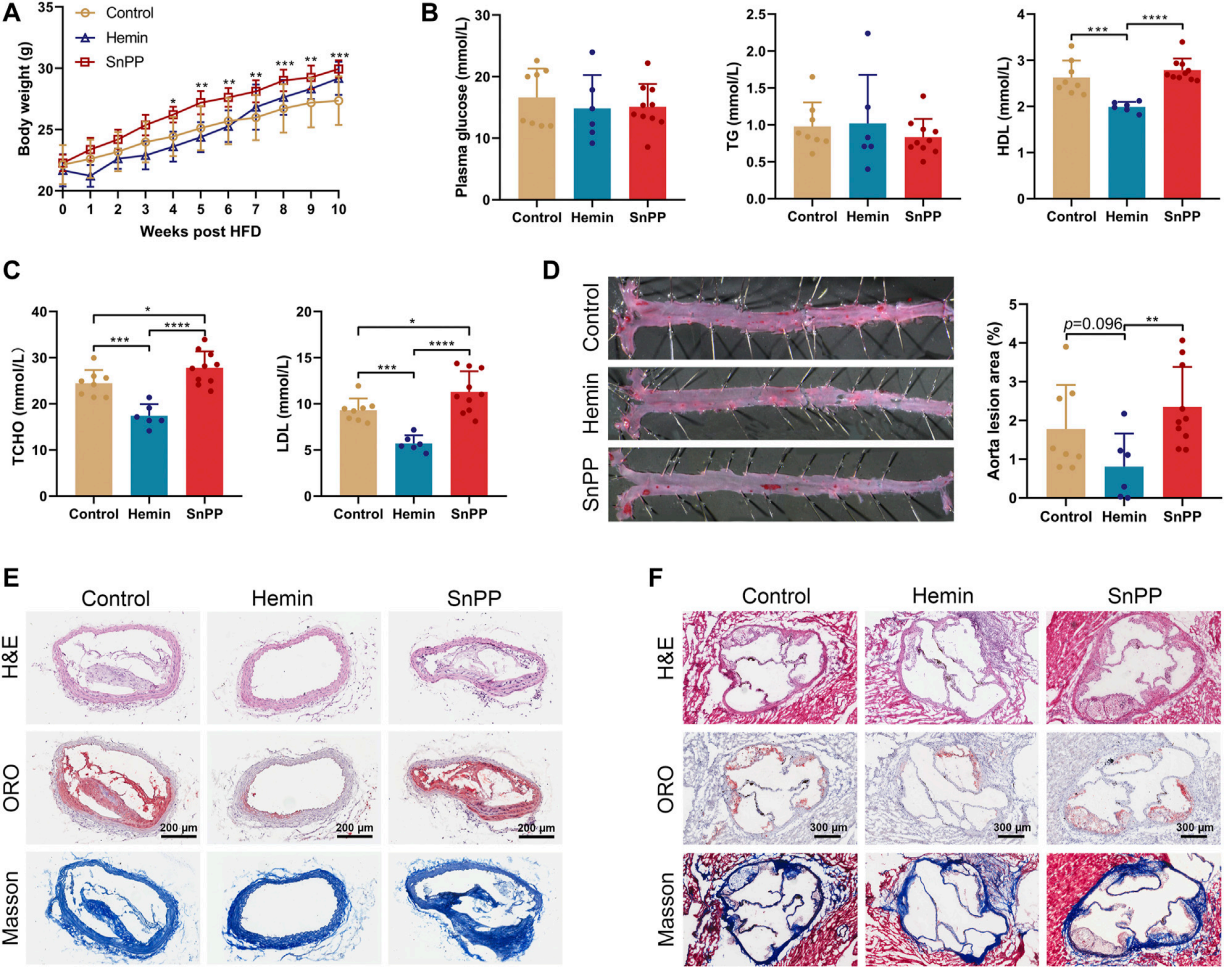
HO-1 inducer hemin ameliorates atherosclerosis in ApoE^-/-^ mice. Male 8-week old ApoE^-/-^ mice fed on a western-type diet (high-fat diet, HFD) were received an intraperitoneal injection of hemin (hemin group, 30 mg/kg/day, *n* = 6), Tin-protoporphyrin IX (SnPP group, 10 mg/kg/day, *n* = 10) and vehicle (control group, *n* = 8) once every other day for 10 weeks. **(A)** Weekly body weight of mice on HFD and under pharmacological challenges. **(B,C)** Plasma lipids profiles. Plasma collected from peripheral blood was subjected to the biochemical analysis of glucose, total triglyceride (TG), high-density lipoprotein cholesterol (HDL), low-density lipoprotein cholesterol (LDL), and total cholesterol (TCHO). (D)Aortas, **(E)** brachiocephalic arteries, and **(F)** aortic roots collected from three groups were subjected to histological and morphometric analysis. **(D)** Representative images of En face oil red O (ORO) staining (left) and quantitative analysis of the ORO (+) staining area over total aorta. Aorta lesions are identified by dark red areas after ORO staining. Data are expressed as mean ± SD. **(E)** Representative images of lesion morphology in brachiocephalic artery. Cells were stained with H&E (upper panel), lipid content (red) was stained with ORO (middle panel), and collagen (blue) was stained with Masson’s Trichrome (lower panel). **(F)** Morphometric and histological analyses of the lesions in aortic sinus/roots. Representative cross-sections of the aortic roots were shown. Cells were stained with H&E (upper panel), lipid content was stained with ORO (middle panel), and collagen was stained with Masson’s Trichrome (lower panel). Data were expressed as mean ± SD. n (control) = 8, n (hemin) = 6, n (SnPP) = 10. *p < 0.05, **p < 0.01, ***p < 0.001, ***p < 0.0001 (One-way ANOVA).

In order to determine whether endogenous HO-1 system is involved in atherogenesis *in vivo*, we treated ApoE^-/-^ mice on a western-type diet with well-proven HO-1 inducer hemin or inhibitor SnPP, respectively. We found that hemin slightly but not significantly decreased the body weight, whereas SnPP significantly increased it (Figure 1A). As assessed by immunostaining in aortic lesions, HO-1 expression was higher in mice treated with hemin, but lower in those treated with SnPP (Supplementary Figure S1A). HO-1 expression in liver was increased by hemin and inhibited by SnPP, indicating a successful induction or inhibition of HO-1 (Supplementary Figure S1B). Hemin or SnPP did not affect blood glucose or triglyceride (Figure 1B), but hemin significantly reduced total cholesterol, LDL cholesterol, and HDL cholesterol levels. Compared to the control group, HO-1 inhibitor SnPP increased the levels of total and LDL cholesterol (Figures 1B,C).

After administration of hemin or SnPP, en face Oil Red O staining was then used to evaluate atherosclerotic lesions in aortas in ApoE^-/-^ mice. As shown in Figure 1D, mice treated with hemin had a smaller lesion area than mice treated with SnPP (*p* = 0.009) or vehicle (*p* = 0.096). The brachiocephalic artery (BCA) is a highly reproducible site of lesion formation and after treatment with hemin, no visible lesions were found in the BCA of mice (Figure 1E). Moreover, hemin-treated mice showed a small atherosclerotic plaque in aortic roots (Figure 1F and Supplementary Figure S1C). Importantly, HO-1 induction by hemin caused a more stable plaque phenotype characterized by reduced lipid content and increased collagen deposition both in BCA and aortic sinus (Figures 1E,F, and Supplementary Figure S1C). Hemin and SnPP did not have a significant effect on AST and ALT activity (Supplementary Figure S1D), suggesting that they do not cause liver damage. These results indicate that HO-1 induction significantly reduces circulating lipid and ameliorates atherogenesis, whereas HO-1 inhibition exacerbates them.

### HO-1 Induction Decreases the Percentage of T and Natural Killer Cells, and Increases the Proportion of Dendritic Cells and Myeloid-Derived Suppressor Cells in the Spleen

**FIGURE 2:**
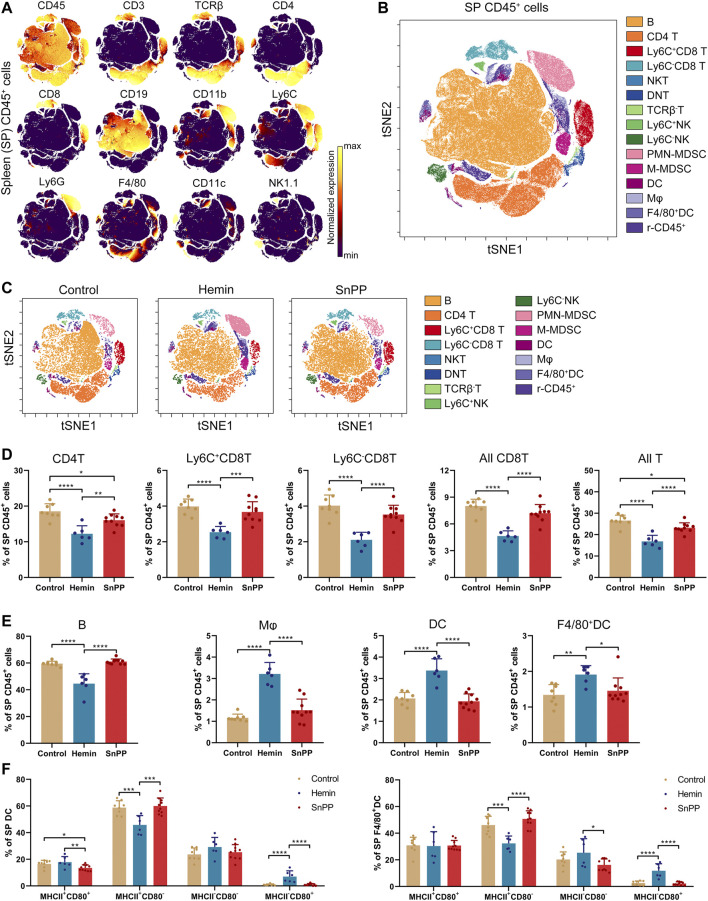
Alterations of immune cell component by HO-1 inducer and inhibitor in the spleen. ApoE^-/-^ mice fed with a western-type diet were treated with hemin (*n* = 6) or SnPP (*n* = 10) or vehicle (control, *n* = 8) for 10 weeks. The spleen (SP) cells were collected from mice and subjected to mass cytometry analysis after staining with 26 metal isotope-labeled antibodies. **(A)** viSNE map showing the distribution of the spleen CD45^+^ immune cells from all three groups. Cells on the viSNE map were colored according to their expression profiles of the indicated surface markers. The color bar indicates the normalized expression of each marker. **(B)** 15 cell populations were identified and colored on the viSNE map. **(C)** Representative viSNE maps of spleen CD45^+^ immune cells from each group. **(D,E)** Bar plots showing the cell frequencies as indicated. **(F)** Bar plots showing the frequencies of indicated cell subsets in spleen DCs (left) or F4/80^+^DCs (right). Only significant changed cell populations are shown. Bar graphs represent mean ± SD. Dots represent individual samples. n (control) = 8, n (hemin) = 6, n (SnPP) = 10. *p < 0.05, **p < 0.01, ***p < 0.001, ****p < 0.0001 (One-way ANOVA). r-CD45, rest of CD45^+^ cells.

**FIGURE 3:**
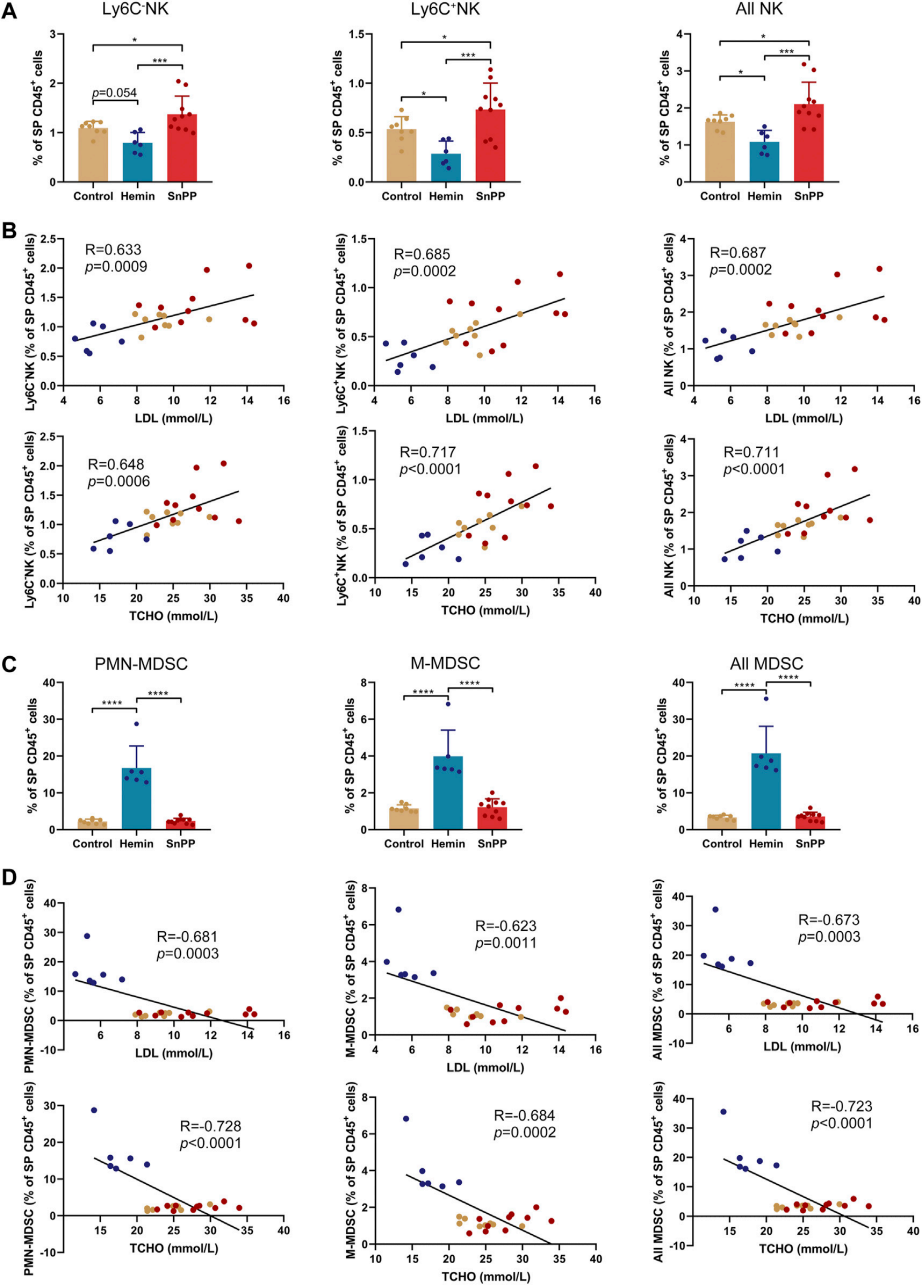
HO-1 inducer and inhibitor affect the NK and MDSC in the spleen. **(A)** Bar plots showing the frequencies of Ly6C^+^NK, Ly6C^−^NK, and all NK cells in spleen (SP) CD45^+^ cells obtained from ApoE^-/-^ mice treated with hemin or SnPP or vehicle, respectively. **(B)** Dot plots (*n* = 24) showing the Pearson correlation coefficients for relationships between the concentrations of total (TCHO) or LDL cholesterol in peripheral blood and the frequencies of indicated NK cell subsets in the spleen. **(C)** Bar plots showing the frequencies of PMN-MDSCs, M-MDSCs, and all MDSCs in spleen CD45^+^ cells obtained from ApoE^-/-^ mice treated with hemin or SnPP or vehicle, respectively. **(D)** Dot plots (*n* = 24) showing the Pearson correlation coefficients for relationships between the concentrations of total (TCHO) or LDL cholesterol in peripheral blood and the frequencies of indicated MDSC subsets in the spleen. Bar graphs represent mean ± SD. Dots represent individual samples. n (control) = 8, n (hemin) = 6, n (SnPP) = 10. *p < 0.05, ***p < 0.001, ****p < 0.0001 (One-way ANOVA).

Modulation of the immune system is closely associated with the progression of atherosclerosis. Since hemin reduced both total and LDL cholesterol in atherosclerosis, we next systemically investigated the immune component of the spleen and peripheral blood using a high-throughput single-cell technique, mass cytometry. After analyzing the 26 surface markers on the spleen cells, viSNE was used to visualize high-dimensional data in two dimensions (Figures 2A–C). Based on the expression profiles of 12 markers (Figure 2A) and phenotypes used for immune cell gating (Supplementary Figure S2A), 15 immune cell populations, including 7 T cell subsets, two NK cell populations, two MDSC subsets, two DC subsets, macrophages, and rest of the CD45^+^ cells, were identified on the viSNE map (Figures 2B,C and Supplementary Figure S2B). The HO-1 inducer, hemin, significantly reduced the proportions of CD4T, Ly6C^+^CD8T, Ly6C^−^CD8T, all CD8T, all T, and B cells (Figures 2D,E). Except for CD4T, all of these cell populations correlated positively with the levels of blood LDL and total cholesterol (Supplementary Figures S2C,D). SnPP, as a HO-1 inhibitor, also slightly decreased CD4T and all T cells without affecting the rest of the T cell subpopulations (Figure 2D). Additionally, hemin increased the percentage of DC, F4/80^+^DC, and macrophages in the spleen, whereas SnPP had no significant effect on these cell populations (Figure 2E). Among these cell populations, DC and macrophage proportions were significantly and negatively related to blood LDL and total cholesterol levels (Supplementary Figure S2D). MHCII and CD80 are markers for DC maturation (Ye et al., 2020). Hemin treatment decreased the proportion of MHCII^+^CD80^−^ cells and increased the proportions of and MHCII^−^CD80^+^ cells in DC and F4/80^+^DC populations, whereas SnPP had no significant effects on these cell sub-population (Figure 2F). The proportions of NK cells, as well as their subsets, Ly6C^+^NK and Ly6C^−^NK, were decreased by hemin treatment, but significantly increased by SnPP (Figure 3A). NK cells and their subsets exhibited moderate (|R|> 0.4) to strong (|R|> 0.7), significant, and positive correlations with LDL and total cholesterol concentrations (Figure 3B). Furthermore, hemin caused a 14.5 and 2.8% increase in the percentages of PMN-MDSCs and M-MDSCs, respectively, whereas SnPP had no effect (Figure 3C). The percentages of PMN-MDSCs and M-MDSCs, as well as all MDSCs, were significantly and negatively related to the concentrations of LDL and total cholesterol with a moderate correlation (|R|> 0.4) (Figure 3D). These results clearly demonstrate the regulatory role of HO-1 induction in spleen immune cells. Hemin significantly regulate NK and MDSCs, which are closely associated with the risk factors for atherosclerosis, suggesting that NK and MDSCs might play a important role in HO-1-induced atherogenesis improvement.

### HO-1 Induction Significantly Affects T Cell Subsets in the Spleen

**FIGURE 4:**
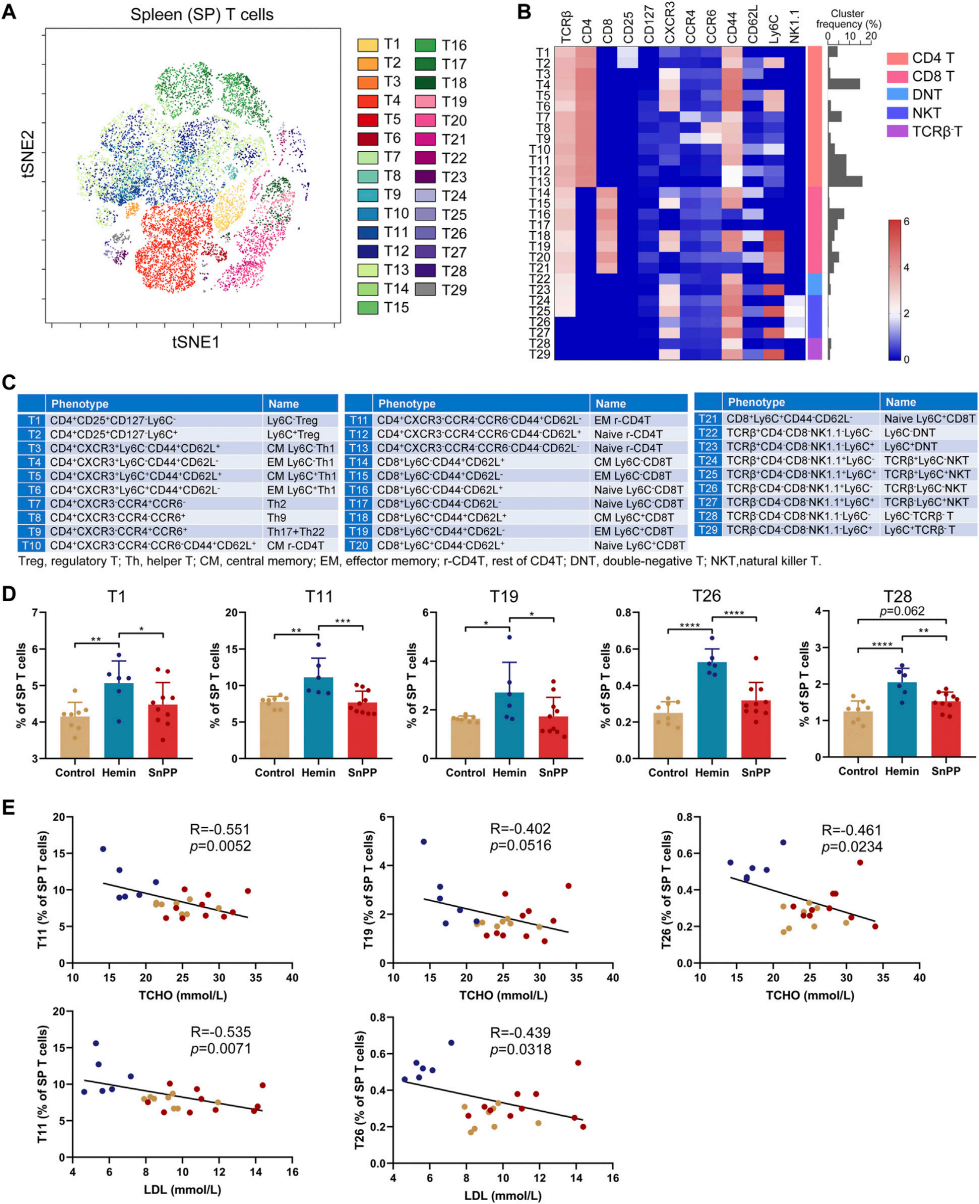
HO-1 inducer increases the proportion of Ly6C^−^Treg, effector memory T and NKT cells in the spleen. After measuring the expression of 26 surface markers on the spleen (SP) cells using mass cytometry, all the T cells (CD3^+^) were analyzed using viSNE. **(A)** viSNE map showing the distribution of the splenic T cells from all three groups. 29 T cell clusters were identified and colored on the viSNE map based on the expression profiles of 12 T cell markers. **(B)** A heatmap showing the normalized expression of 12 indicated markers in 29 T cell clusters. **(C)** Phenotypes and names of 29 T cell clusters. **(D)** Bar plots showing the frequencies of T1, T11, T19, T26, and T28 in spleen. **(E)** Dot plots (*n* = 24) showing the Pearson correlation coefficients for relationships between the concentrations of blood total (TCHO) or LDL cholesterol and the frequencies of T11, T19, or T26. Only significant changed T cell clusters and correlationships with |R|>0.4 and *p* < 0.05 are shown. Bar graphs represent mean ± SD. n (control) = 8, n (hemin) = 6, n (SnPP) = 10. *p < 0.05, **p < 0.01, ***p < 0.001, ****p < 0.0001 (One-way ANOVA). Correlations were determined by a Pearson test.

**FIGURE 5:**
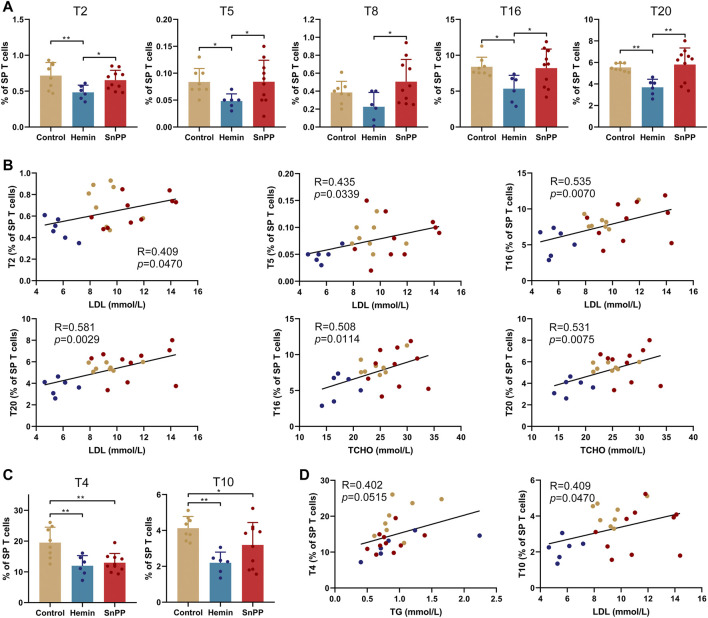
HO-1 inducer decreases Ly6C^+^Treg, central memory Ly6C^+^Th1 and naive CD8T cells in the spleen. **(A)** Bar plots showing the frequencies of T2, T5, T8, T16, and T20 in spleen (SP) T cells obtained from ApoE^-/-^ mice treated with hemin or SnPP or vehicle, respectively. **(B)** Dot plots (*n* = 24) showing the Pearson correlation coefficients for relationships between the concentrations of blood total (TCHO) or LDL cholesterol and the frequencies of T2, T5, T16, or T20. **(C)** Bar plots showing the frequencies of T4 and T10 in spleen T cells obtained from ApoE^-/-^ mice treated with hemin or SnPP or vehicle. **(D)** Dot plots (n = 24) showing the Pearson correlation coefficients for relationships between the concentrations of blood triglyceride (TG) or LDL cholesterol and the frequencies of T4 or T10. Only significant changed T cell clusters and correlationships with |R|>0.4 and *p* < 0.05 are shown. Bar graphs represent mean ± SD. n (control) = 8, n (hemin) = 6, n (SnPP) = 10. *p < 0.05, **p < 0.01 (One-way ANOVA). Correlations were determined by a Pearson test.

Inflammation is largely modulated by T cells and we next comprehensively analyzed the effect of the HO-1 pathway on spleen T clusters using 12 markers and viSNE. Based on the expression patterns of these 12 markers on T cells (Supplementary Figure S3A), 29 T cell sub-populations, including 13 CD4 T, eight CD8 T, 2 double-negative T (DNT), 4 NKT, and 2 TCRβ^−^ T cell clusters, were identified in spleen T cells (Figures 4A–C). Their phenotypes and name were displayed in Figure 4C. The proportions of T1 (Ly6C^−^Treg), T11 (rest of effector memory CD4 T, EM r-CD4T), T19 (EM Ly6C^+^CD8T), T26 (TCRβ^−^Ly6C^−^NKT), and T28 (Ly6C^−^TCRβ^-^T) were significantly increased by hemin treatment, while SnPP did not affect these clusters (Figure 4D). Among them, T11 and T26 were significantly and negatively correlated with the levels of LDL and total cholesterol. T19 displayed a negative correlation with total cholesterol levels with a trend close to significant (*p* = 0.0516) (Figure 4E). Hemin also dramatically decreased T2 (Ly6C^+^Treg), T5 (central memory Ly6C^+^Th1, CM Ly6C^+^Th1), T16 (naive Ly6C^−^CD8T), and T20 (naive Ly6C^+^CD8T), but no such effect was observed with SnPP (Figure 5A). Among these clusters, T2, T5, T16, and T20 were positively correlated with LDL concentrations in the blood. T16 and T20 were also positively correlated with total cholesterol levels in the blood (Figure 5B). Both hemin and SnPP increased the percentage of T9 (Th17 + Th22), T13 (naive r-CD4T), T22 (Ly6C^−^DNT), and T24 (TCRβ^+^Ly6C^−^NKT), and decreased the proportion of T3 (CM Ly6C^−^Th1) and T4 (EM Ly6C^−^Th1, Figure 5C and Supplementary Figure S3B). The percentage of T15 (EM Ly6C^−^CD8T) and T10 (CM r-CD4T) was decreased by SnPP and hemin, respectively (Figure 5C and Supplementary Figure S3B). Among these clusters, T4 and T10 showed positive correlations with blood triglyceride levels and LDL levels, respectively (Figure 5D). The rest of the T cell clusters were not affected by either hemin or SnPP. The data suggest that hemin has a significant role in regulating T cells and HO-1 induction can reshape the T cell components in the spleen, improving blood lipid metabolism and reducing atherosclerosis.

### HO-1 Induction Reduces the Proportion of T, B, and NK Cells, and Increases the Proportion of DCs and MDSCs in the Peripheral Blood

**FIGURE 6:**
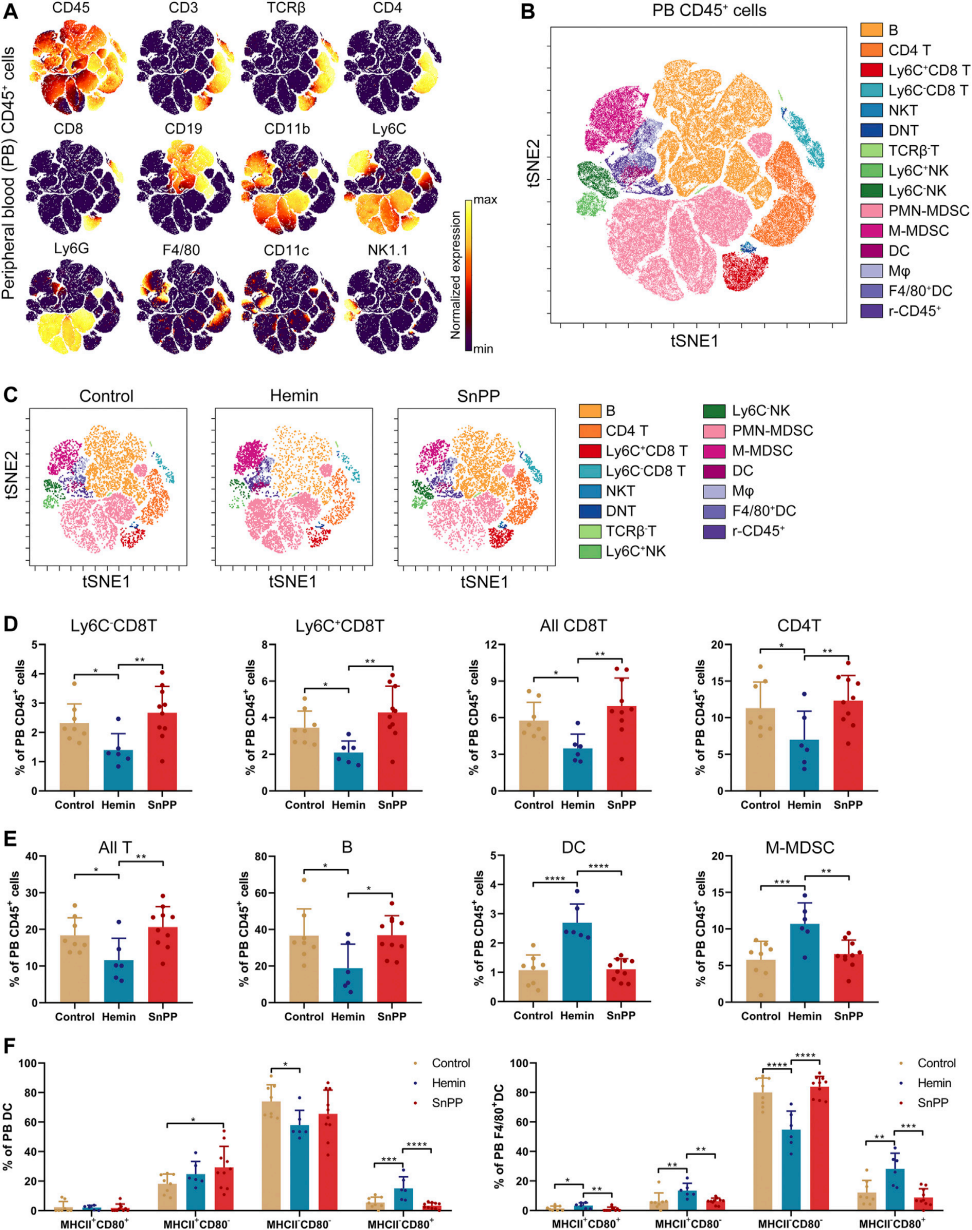
Hemin and SnPP differently alter the immune cell populations in peripheral blood. ApoE^-/-^ mice fed with a western-type diet were treated with hemin (*n* = 6) or SnPP (*n* = 10) or vehicle (control, *n* = 8) for 10 weeks. The peripheral blood (PB) cells were collected and subjected to mass cytometry analysis after staining with 26 metal isotope-labeled antibodies. **(A)** viSNE map showing the distribution of the PB CD45^+^ immune cells from all three groups. Cells on the viSNE map were colored according to the expression profiles of indicated surface markers. The color bar indicates the normalized expression of each marker. **(B)** 15 cell populations were identified and colored on the viSNE map. **(C)** Representative viSNE maps of PB CD45^+^ immune cells from each group. **(D,E)** Bar plots showing the frequencies of the indicated cell populations and sub-clusters. **(F)** Bar plots showing the frequencies of indicated subsets in PB DCs and F4/80^+^DCs. Only significant changed cell populations are shown. Bar graphs represent mean ± SD. Dots represent individual samples. n (control) = 8, n (hemin) = 6, n (SnPP) = 10. *p < 0.05, **p < 0.01, **p < 0.01, ***p < 0.001, ****p < 0.0001 (One-way ANOVA).

We further comprehensively analyzed the immune cell changes in the peripheral blood. Similar to spleen, 15 main immune cell populations were identified in the peripheral blood (Figures 6A–C, and Supplementary Figure S4A). Similar to the results observed in the spleen, hemin decreased the proportions of CD4 T, two CD8 T subsets, all T cells, and B cells (Figures 6D,E). All of them except CD4T were significantly and positively correlated with the levels of LDL and total cholesterol (Supplementary Figures S4B,C). Meanwhile, hemin increased the percentages of DCs and M-MDSCs (Figure 6E), and DC levels were correlated negatively with the levels of blood LDL and total cholesterol (Supplementary Figure S4C). Hemin treatment resulted in a significant decrease in the proportion of MHCII^−^CD80^−^ cells, but an increase in MHCII^−^CD80^+^ cells in both DC and F4/80^+^DCs (Figure 6F). In F4/80^+^DCs, hemin increased the proportion of both MHCII^+^CD80^+^ and MHCII^+^CD80^−^ cells. Except for the percentage of MHCII^+^CD80^−^ cells in DCs, no significant alternations in the other subsets were observed with SnPP treatment (Figure 6).

**FIGURE 7:**
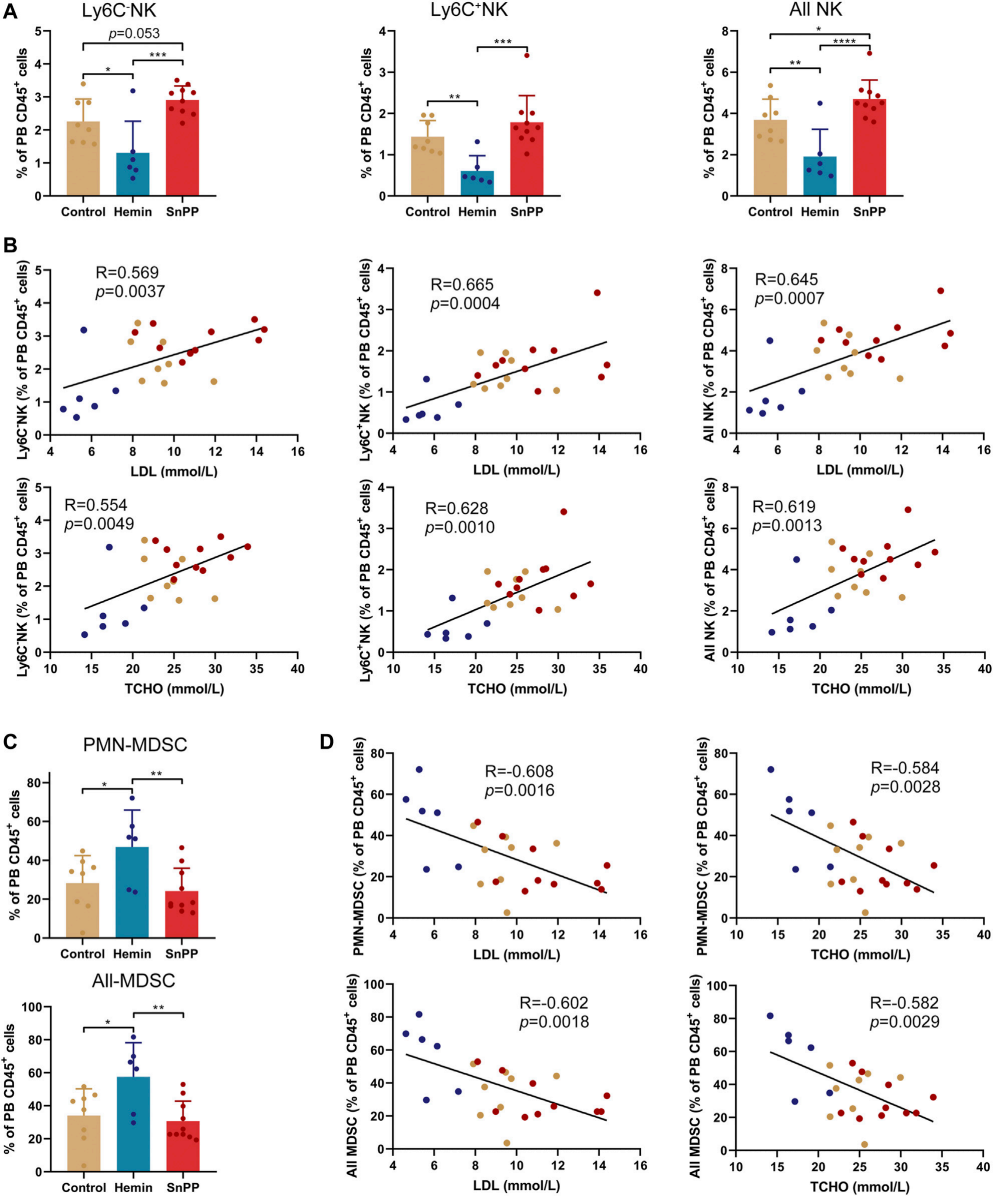
HO-1 inducer and inhibitor significantly affect the peripheral NK and MDSCs. **(A)** Bar plots showing the frequencies of Ly6C^−^NK, Ly6C^+^NK, and all NK cells in peripheral blood (PB) CD45^+^ cells with various treatments as indicated. **(B)** Dot plots (*n* = 24) showing the Pearson correlation coefficients for relationships between the concentrations of total (TCHO) or LDL cholesterol in peripheral blood and the frequencies of indicated NK cell subsets in PB CD45^+^ cells. **(C)** Bar plots showing the frequencies of PMN-MDSCs and all MDSCs in PB CD45^+^ cells obtained from ApoE^-/-^ mice treated with hemin or SnPP or vehicle, respectively. **(D)** Dot plots (*n* = 24) showing the Pearson correlation coefficients for relationships between the concentrations of blood total (TCHO) or LDL cholesterol and the frequencies of indicated MDSC subsets in PB CD45^+^ cells. Bar graphs represent mean ± SD. Dots represent individual samples. n (control) = 8, n (hemin) = 6, n (SnPP) = 10. *p < 0.05, **p < 0.01, ***p < 0.001, ****p < 0.0001 (One-way ANOVA).

Moreover, hemin significantly decreased Ly6C^−^NK, Ly6C^+^NK, and all NK cells, whereas SnPP increased them with either significant or a trend close to significant except for Ly6C^+^NK cells (Figure 7A). The percentage of NK cells, as well as their two subsets, displayed significant and positive correlations with blood LDL and total cholesterol levels (Figure 7B). Hemin also increased the proportions of PMN-MDSCs and all MDSCs, which were negatively correlated with blood LDL and total cholesterol concentrations (Figures 7C,D). All of these data suggest that HO-1 induction can significantly reshape peripheral immune cells, especially NK and MDSCs, which are closely associated with atherosclerosis risk factors.

### HO-1 Induction Moderately Affects T Cell Subsets in Peripheral Blood

**FIGURE 8:**
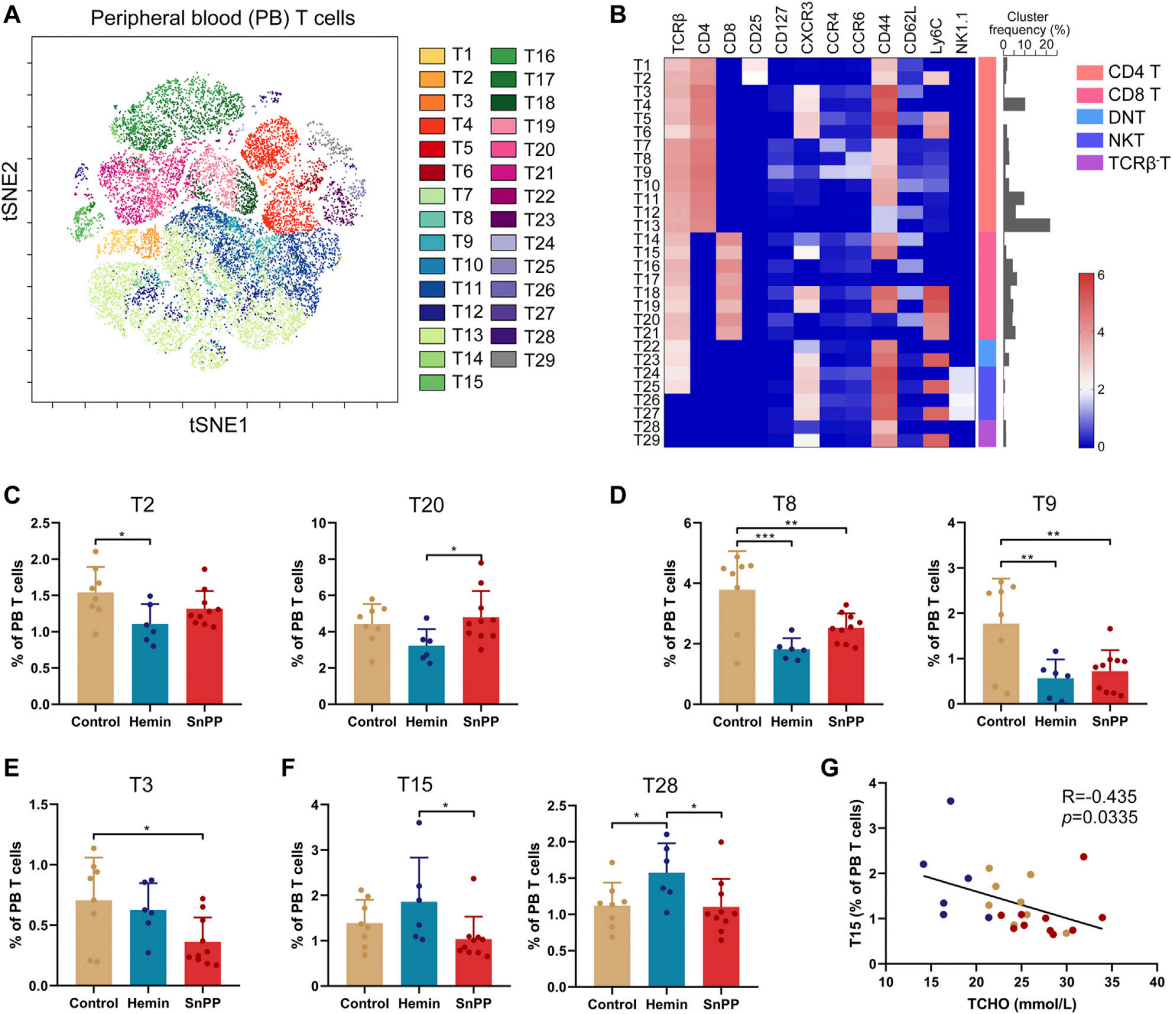
Hemin and SnPP moderately change T cell clusters in peripheral blood. After measuring the expression of 26 surface markers on the peripheral blood (PB) cells using mass cytometry, all the T cells (CD3^+^) were analyzed using viSNE. **(A)** 29 T cell clusters were identified and colored on the viSNE map based on the expression patterns of 12 T cell markers. **(B)** A heatmap showing the normalized expression of 12 indicated markers in 29 T cell clusters. **(C–F)** Bar plots showing the frequencies of the indicated T cell sub-clusters in PB T cells isolated from ApoE^-/-^ mice treated with hemin or SnPP or vehicle, respectively. **(G)** Dot plots (*n* = 24) showing the Pearson correlation coefficients for relationships between the concentrations of blood total cholesterol (TCHO) and the frequency of T15 clusters. Only significant changed T cell clusters and correlationships with |R|>0.4 and *p* < 0.05 are shown. Bar graphs represent mean ± SD. n (control) = 8, n (hemin) = 6, n (SnPP) = 10. *p < 0.05, **p < 0.01, ***p < 0.001 (One-way ANOVA). Correlations were determined by a Pearson test.

The peripheral blood T cells were then further analyzed by viSNE (Figure 8A and Supplementary Figure S5A) and 29 T cell clusters were identified (Figure 8B) according to the phenotypes of T cell clusters (Figure 4C). However, only four clusters, including T2 (Ly6C^+^Treg), T8 (Th9), T9 (Th17 + Th22), and T28 (Ly6C^−^TCRβ^-^T), were dramatically affected by hemin. Specifically, hemin treatment significantly decreased T2, T8, and T9, but increased T28 (Figures 8C–F). On the other hand, T3, T8, and T9 were significantly decreased by SnPP (Figures 8D,E). T3 (CM Ly6C^−^Th1) was correlated negatively with blood LDL and total cholesterol levels (Supplementary Figure S5B), while T15 (EM Ly6C^−^CD8T) was correlated negatively with blood total cholesterol (Figure 8G). The rest of the T cell clusters were not significantly affected by either hemin or SnPP. Taken together, hemin or SnPP had only moderate effects on peripheral blood T cells, suggesting that peripheral blood T cells may play a minor role in HO-1-mediated atherogenesis.

## Discussion

Due to its antioxidant and anti-inflammatory properties, HO-1 provides protection against atherosclerosis and other cardiovascular diseases (Campbell et al., 2021; Durante, 2011). As an HO-1 inducer, hemin attenuates atherosclerotic lesion formation in LDLr deficient mice by inhibiting lipid peroxidation and affecting the nitric oxide pathway (Ishikawa et al., 2001b). A deficiency of HO-1 accelerates and exacerbates atherosclerotic lesion formation (Yet et al., 2003). Immunity, including monocytes/macrophages, dendritic cells, T cells, NK cells, mast cells, platelets, and a number of inflammatory mediators and pathways, are involved in the initiation, progression, and rupture of atherosclerotic plaques. Using HO-1 inducer and inhibitor, we confirmed the protective role of HO-1 in murine atherosclerosis and demonstrated its profound effect on systemic immunity using mass cytometry-based single-cell analysis. Through in-depth analyses, we discovered that the HO-1 inducer hemin effectively ameliorates atherosclerosis by lowering circulating cholesterol levels and reshaping immune cells in the spleen and peripheral blood. Our data further support HO-1 as a promising therapeutic target in atherosclerosis treatment.

Oxidized LDL is a major determinant in the pathogenesis of atherosclerosis since it is taken up by immune cells attaching to the injured (or activated) artery wall, leading to the transformation into foam cells and initiation of atherosclerotic plaques (Libby et al., 2002). A previous study have shown that hemin decreased the plasma lipid hydroperoxide levels and SnPP increased them in LDLr^-/-^ mice (Ishikawa et al., 2001b). Byproducts of HO-1, such as biliverdin, bilirubin, and CO, function as antioxidants by scavenging oxygen free radicals and organic peroxy radicals. Among them, bilirubin protects cells from lipid-induced damage and prevents LDL peroxidation (Stocker et al., 1987; Boon et al., 2012), thereby slowing down the process of atherosclerosis. After hemin administration, they are catalyzed by HO-1 to generate biliverdin, carbon monoxide (CO), and ferrous iron (Verheij et al., 2021). Biliverdin is subsequently transformed into bilirubin. Since biliverdin and bilirubin are antioxidants, the anti-atherogenic properties of hemin is likely mediated by these byproducts.

The innate immune response as well as the adaptive immune response are critical for the pathogenesis of atherosclerosis. Macrophages play a major role in eliminating oxidized LDL, while excessive LDL uptake induced apoptosis (Elliott et al., 2017). An increase in apoptotic macrophages in the plaques results in secondary necrosis and thereafter the formation of a necrotic core (Martinet et al., 2011; Gonzalez and Trigatti, 2017). A reduction in circulating macrophages reduces the clearance of oxidized LDL, which can exacerbate atherosclerotic lesion formation. In our study, we found that hemin treatment resulted in an increase of macrophages in the spleen by more than three times, suggesting an increased capacity for removing LDL. Indeed, our data showed that increased spleen macrophages were associated with lower levels of total and LDL cholesterol in the blood.

As a link between the innate and adaptive immune systems, DCs are crucial to the artery-specific immune response. These cells either promote or inhibit atherogenesis through regulating lipid uptake, antigen presentation, and cytokine secretion (Herrero-Fernandez et al., 2019; Jongstra-Bilen et al., 2006; Liu et al., 2008). DCs can protect against atherosclerosis by activating Treg cells, which suppress the inflammatory response (Choi et al., 2011). Here, we observed a significant increase in DCs in both the spleen and peripheral blood after hemin treatment. Moreover, hemin increased the proportion of T1 cells, which are Ly6C^−^Treg cells, in the spleen. Since DCs can induce Treg cells, hemin-induced DCs may be responsible for the increase in Treg cells. An inflammatory environment promotes the maturation of dendritic cells along with the upregulation of MHCII and co-stimulatory molecules (Durante, 2011). Maturate DCs present antigens to T cells through surface MHCII and activate them through co-stimulatory molecules. Although the proportions of DCs and F4/80^+^DCs were increased by hemin in the spleen, the MHCII^+^ cells in these two DC subsets were reduced, suggesting that hemin attenuates their capacity to present antigens and therefore reduces the T cell-mediated inflammation. In the spleen, the proportion of T cells, including CD4 T and CD8 T cells, was significantly decreased by hemin treatment, possibly due to the decreased ability of DCs to present antigens. DCs found in peripheral blood are immature because they lack MHCII and co-stimulatory molecule CD80, making them incapable of presenting antigens. Furthermore, the proportion of DCs in both the spleen and peripheral blood was negatively correlated with the levels of blood total and LDL cholesterol, indicating that hemin-induced DCs may contribute to the decrease in circulating cholesterol.

NK cells, derived from common bone marrow progenitor cells, are characterized by their ability to eliminate aberrant cells without any priming or prior activation. These cells exhibit both innate and adaptive immune features and present a critical component of the innate immune system (van Erp et al., 2020; Yang et al., 2021). As revealed by single-cell RNA sequencing- and mass cytometry-based single-cell analysis, NK cells have been detected in human atherosclerotic plaques and mouse atherosclerotic aorta (Fernandez et al., 2019; Winkels et al., 2018). The accumulation of NK cells in atherosclerotic lesions leads to the expansion of necrotic cores and accelerates atherosclerosis through the release of perforin, granzyme B, and pro-inflammation factor IFN-γ (Selathurai et al., 2014). It is well documented that the HO-1 pathway regulates inflammation in atherosclerosis *via* either direct or indirect effects on a variety of immune cells, including macrophages, DCs, Th1, Th17, Treg, and monocytes (Campbell et al., 2021; Durante, 2011). However, the direct connection between NK cells and the HO-1 pathway in atherosclerosis has not yet been reported. After hemin treatment, we observed a significant decrease in NK cells in both the spleen and peripheral blood as well as a reduction in atherosclerosis. In previous studies, depletion of NK cells by anti-Asialo-GM1 antibody decreased atherosclerosis in ApoE^-/-^ mice (Selathurai et al., 2014) and transgenic elimination of functional NK cells also ameliorated atherosclerosis in LDLr^-/-^ mice (Whitman et al., 2004). NK cells and circulating cholesterol have a clear positive correlation, so a decrease in NK cells caused by hemin may directly contribute to the relief of atherosclerosis symptoms. Meanwhile, SnPP, as a HO-1 inhibitor, persistently and exclusively increased the NK cells in both the spleen and peripheral blood, and exacerbated atherosclerotic lesion in ApoE^-/-^ mice, further highlighting the contribution of NK cells to atherosclerosis. A previous study has shown that the elevated NK cells *via* adoptive transfer doubled atherosclerotic lesion size in ApoE^-/-^Rag2^-/-^IL2rg^-/-^ mice (Whitman et al., 2004), indicating that SnPP can exacerbate atherosclerosis through NK cells. This previous evidence, together with ours, highlights the critical role of NK cells in the pathogenesis of atherosclerosis. Importantly, our findings propose that the protective role of HO-1 in atherosclerosis may be closely associated with the regulation of NK cells as its inducer (hemin) and inhibitor (SnPP) oppositely affect NK cells and atherosclerosis.

MDSCs represent a heterogeneous population of immature myeloid cells and negatively regulate the immune response through producing immunosuppressive factors, including IL-10, transforming growth factor-β, inducible nitric oxide synthase, and reactive oxygen species (De Veirman et al., 2014). As a result of MDSC activation, T cell activation and proliferation, antigen recognition, and NK cell cytotoxicity are suppressed, and regulatory T cells are induced, eventually leading to immunosuppression (Gabrilovich and Nagaraj, 2009). In mice, expression of both Gr-1 (Ly6C/Ly6G) and CD11b are widely used to define MDSCs. Two subsets of MDSCs, PMN-MDSC (CD11b^+^Ly6G^+^Ly6C^lo^) and M-MDSC (CD11b^+^Ly6G^−^Ly6C^hi^), are classified according to their different phenotypic and morphological features (Bronte et al., 2016; Wang et al., 2016). MDSCs have been implicated in chronic inflammation-related diseases, including obesity and atherosclerosis (Foks et al., 2016; Xia et al., 2011). Because of their potent immunosuppressive function, adoptive transfer of MDSCs has been shown to significantly alleviate atherosclerosis through suppression of T cell-mediated pro-inflammatory immune responses (Foks et al., 2016). A six-fold increase in spleen MDSCs and an apparent increase of peripheral blood MDSCs were observed in mice treated with hemin, implying an enhanced immunosuppression by hemin. Elevated MDSCs by hemin in both spleen and peripheral blood was accompanied by lower cholesterol, supporting the role of MDSCs in atherosclerosis. Several studies have reported that MDSCs, induced by endotoxin or histone deacetylase inhibitor or baicalein, inhibit immune responses *via* HO-1 (De Wilde et al., 2009; Rosborough et al., 2012; Li et al., 2019). Here, we provided *in vivo* evidence for the important and effective role of HO-1 in MDSC induction and anti-inflammatory effects in atherosclerosis. Despite many changes in immune cell composition in mice treated with hemin, the precise mechanisms driving these changes remain unknown. Therefore, future investigations will need to provide mechanistic insights into how systemic immunity is remodeled by the HO-1 pathway to enable the development of novel therapeutic strategies for atherosclerosis.

In summary, we confirmed the critical regulatory role of the HO-1 pathway in reshaping systemic immunity in the context of atherosclerosis. Importantly, using mass cytometry-based single cell analysis, we thoroughly dissected the profiles of immune cell lineages and respective cell subsets modulated by hemin- and SnPP in both spleen and peripheral blood. This study increases our understanding of the HO-1 pathway, and provides a potential new strategy to treat atherosclerosis by targeting HO-1.

## Data Availability

The original contributions presented in the study are included in the article/Supplementary Material, further inquiries can be directed to the corresponding authors.
