# Supplementary material for: Induction of Heme Oxygenase-1 Modifies the Systemic Immunity and Reduces Atherosclerotic Lesion Development in ApoE Deficient Mice
Source: Front Pharmacol. 2022 Feb 24;13:809469. doi: 10.3389/fphar.2022.809469 (PMC8908104; doi:10.3389/fphar.2022.809469)
Supplement: Supplementary file 1 [file DataSheet1.PDF]

Supplementary Material

Supplemental methods

Immunofluorescence staining

The distribution of HO-1 in aortic sinus lesions were determined by staining with rabbit anti–heme oxygenase 1 (HO-1) antibody (ab68477) and detecting with donkey anti-rabbit IgG H&L (Alexa Fluor 488) antibodies (ab150073). Nuclei were counterstained with DAPI. All antibodies were purchased from Abcam.

Western blot

Livers were collected and homogenized in ice-cold RIPA buffer. Lysates were maintained constant agitation for 2 hours at 4 °C. Gently aspirate the supernatant to a prechilled tube after the centrifugation at 13,200 rpm for 10 min at 4°C. The protein level of lysates was determined by the BCA protein assay. An aliquot of proteins was separated by SDS-PAGE and then transferred to the immunoblot PVDF membrane. The immunoblots were incubated with primary antibodies at 4 °C overnight, followed by HRP-conjugated secondary antibodies (1:5000) at RT for 1 hour. The signals of targeted proteins were visualized by employing the ECL detection system (Amersham). Primary antibodies used for immunoblots were rabbit polyclonal antibodies against HO-1 (ab68477 diluted 1:1000). All antibodies were purchased from Abcam.

Supplemental Figures

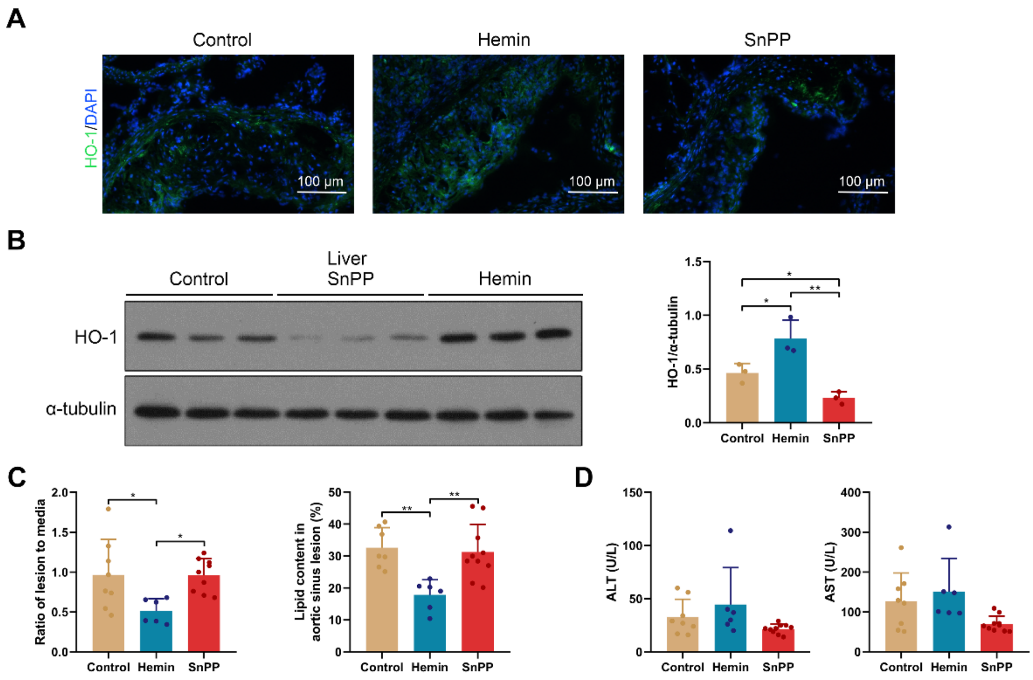

Figure S1. Hemin induces HO-1 expression in aortic lesions and livers of ApoE$^{-/-}$ mice (A) ApoE$^{-/-}$ mice were treated with hemin, SnPP, or vehicle for 10 weeks. Representative images of HO-1 expression (green) in aortic lesions analyzed by immunofluorescence staining and nucleus were counterstained with DAPI (blue). (B) HO-1 expressions in liver were determined by western blot and bar graph showed HO-1/α-tubulin band density ratios. (C-D) Male 8-week old ApoE-/- mice fed on a western-type diet were received an intraperitoneal injection of hemin (hemin group, 30 mg/Kg/day, n=6), Tin-protoporphyrin IX (SnPP group, 10mg/Kg/day, n=10) and vehicle (control group, n=8) once every other day for ten weeks. (C) Quantitative analysis of the ratios of intimal lesion to media of aortic sinus/roots from three groups. The lipid content in aortic sinus lesion was also quantified. (D) Concentrations of liver enzymes (AST&ALT) were determined by biochemical analysis. Bar graphs represent mean ± SD. *$P < 0.05$ (One-way ANOVA).

A

Phenotype	Population
CD45$^+$CD3$^-$CD19$^+$CD11c$^-$	B
CD45$^+$CD3$^+$TCRβ$^+$CD8$^-$CD4$^+$	CD4 T
CD45$^+$CD3$^+$TCRβ$^+$CD8$^+$CD4$^-$Ly6C$^+$	Ly6C$^+$CD8 T
CD45$^+$CD3$^+$TCRβ$^+$CD8$^+$CD4$^-$Ly6C$^-$	Ly6C$^-$CD8 T
CD45$^+$CD3$^+$CD8$^-$CD4$^-$NK1.1$^+$	NKT
CD45$^+$CD3$^+$TCRβ$^+$CD8$^-$CD4$^-$	DNT
CD45$^+$CD3$^+$TCRβ$^-$CD8$^-$CD4$^-$NK1.1$^-$	TCRβ$^-$T
CD45$^+$CD3$^-$NK1.1$^+$Ly6C$^+$	Ly6C$^+$NK
CD45$^+$CD3$^-$NK1.1$^+$Ly6C$^-$	Ly6C$^-$NK
CD45$^+$CD11b$^+$Ly6G$^+$	PMN-MDSC
CD45$^+$CD11b$^+$Ly6C$^+$	M-MDSC
CD45$^+$CD11b^{low}F4/80$^-$CD11c$^+$	DC
CD45$^+$F4/80$^+$CD11c$^-$	Mφ
CD45$^+$F4/80$^+$CD11c$^+$	F4/80$^+$DC

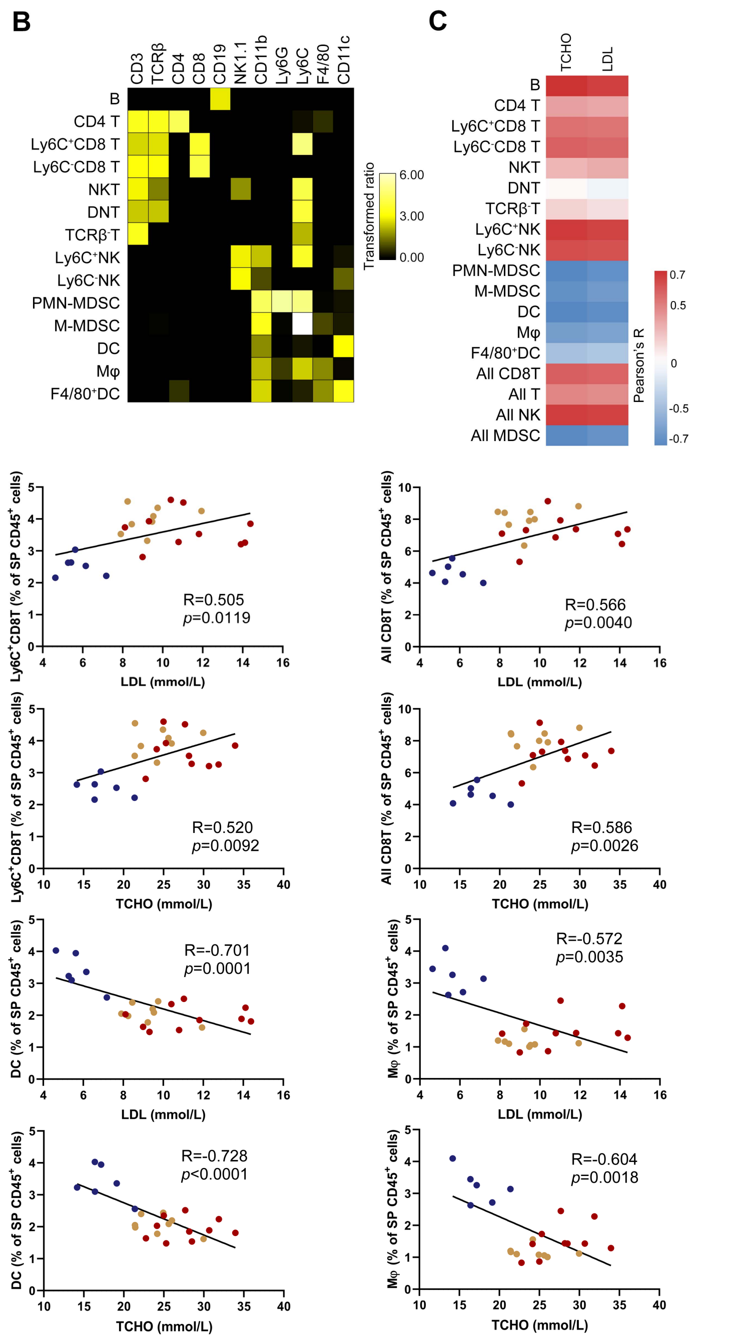

Figure S2. Multiple immune cells in the spleen were correlated with the blood lipids. (A) Phenotypes of 14 immune cell populations. (B) A heatmap showing the normalized expression of 11 indicated markers in 14 spleen cell populations. (C) A heatmap showing the Pearson correlation coefficients for relationships between the concentrations of TCHO or LDL in peripheral blood and the frequencies of indicated spleen cell populations. (D) Dot plots (n=24) showing the Pearson correlation coefficients for relationships between the concentrations of total (TCHO) or LDL cholesterol in peripheral blood and the frequencies of indicated cell populations in the

spleen. Correlations were determined by a Pearson test. Only correlationships with |R|>0.4 and p<0.05 are shown.

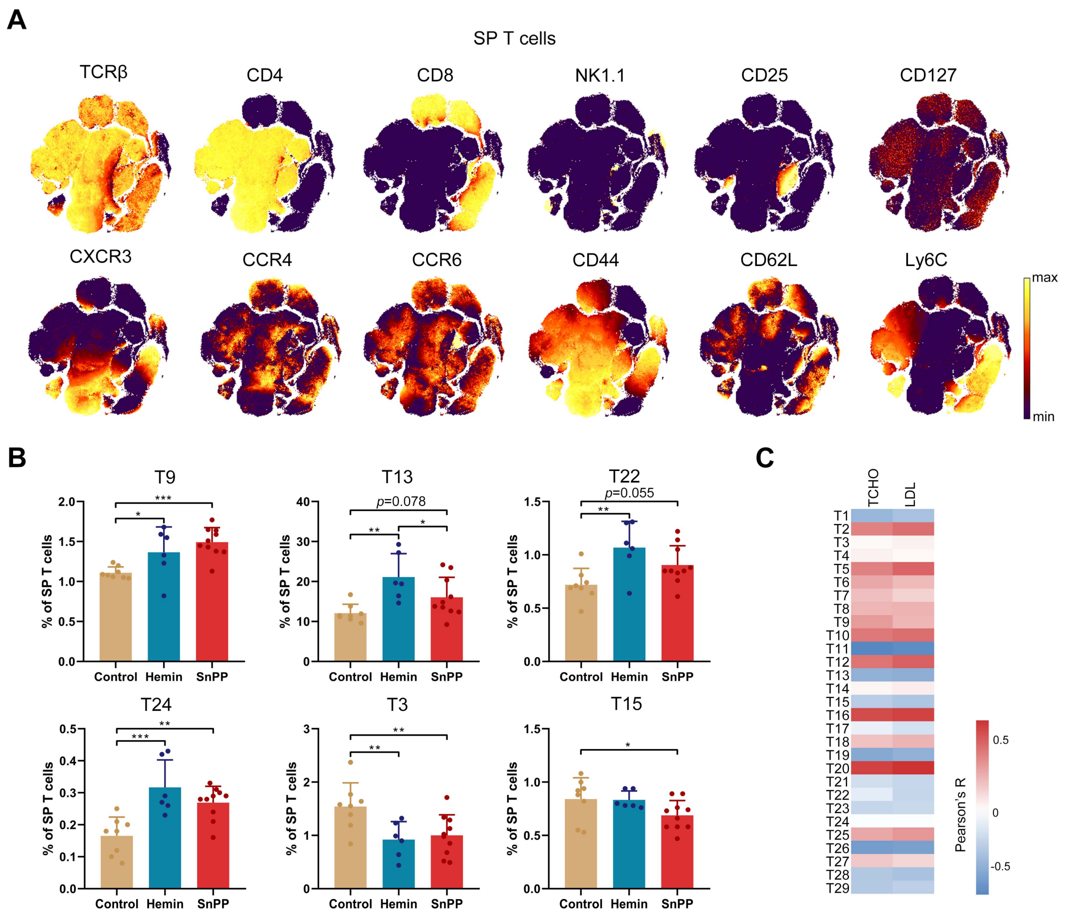

Figure S3. HO-1 inducer and inhibitor changed multiple T cells in the spleen. (A) viSNE map showing the distribution of the total splenic T cells from three groups. Cells on the viSNE map were colored by the normalized expression of indicated surface markers. (B) Bar plots showing the frequencies of the indicated T cell sub-populations in spleen treated with hemin or SnPP or vehicle, respectively. (C) A heatmap showing the Pearson correlation coefficients for relationships between the concentrations of TCHO or LDL in peripheral blood and the frequencies of indicated spleen T cell clusters. Only significant changed T cell clusters are shown. Bar graphs represent mean ± SD. *$P < 0.05$, **$P < 0.01$, ***$P < 0.001$ (One-way ANOVA). Correlations were determined by a Pearson test.

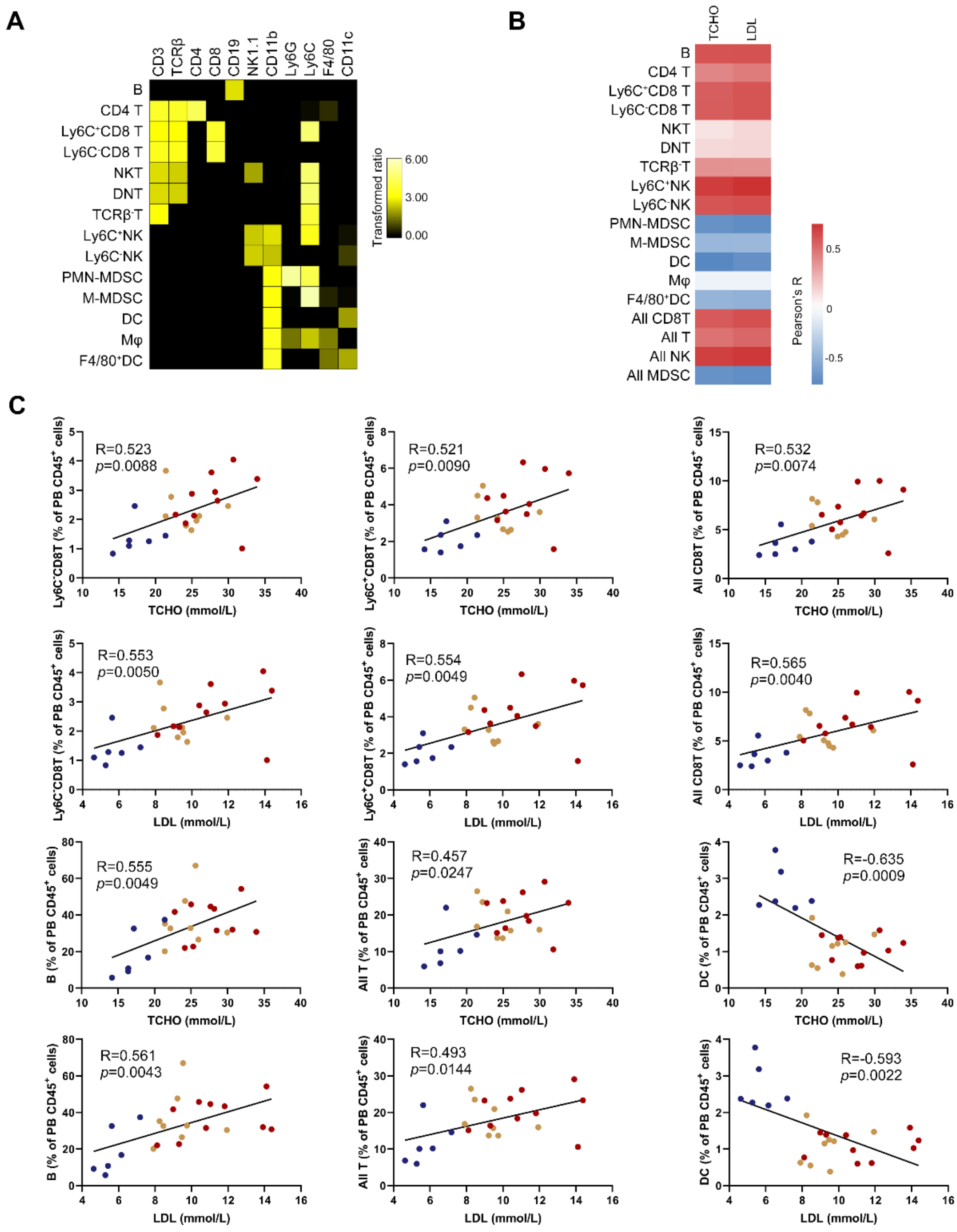

Figure S4. Multiple immune cells in the peripheral blood (PB) correlate with the blood lipids. (A) A heatmap showing the normalized expression of 11 indicated markers in 14 PB cell populations. (B) A heatmap showing the Pearson correlation coefficients for relationships between the concentrations of TCHO or LDL in peripheral blood and the frequencies of indicated PB cell populations. (C) Dot plots (n=24) showing the Pearson correlation coefficients for relationships between the concentrations of total (TCHO) or LDL cholesterol in peripheral blood and the frequencies of indicated cell populations in the PB. Correlations were determined by a

Pearson test. Only correlationships with |R|>0.4 and p<0.05 are shown.

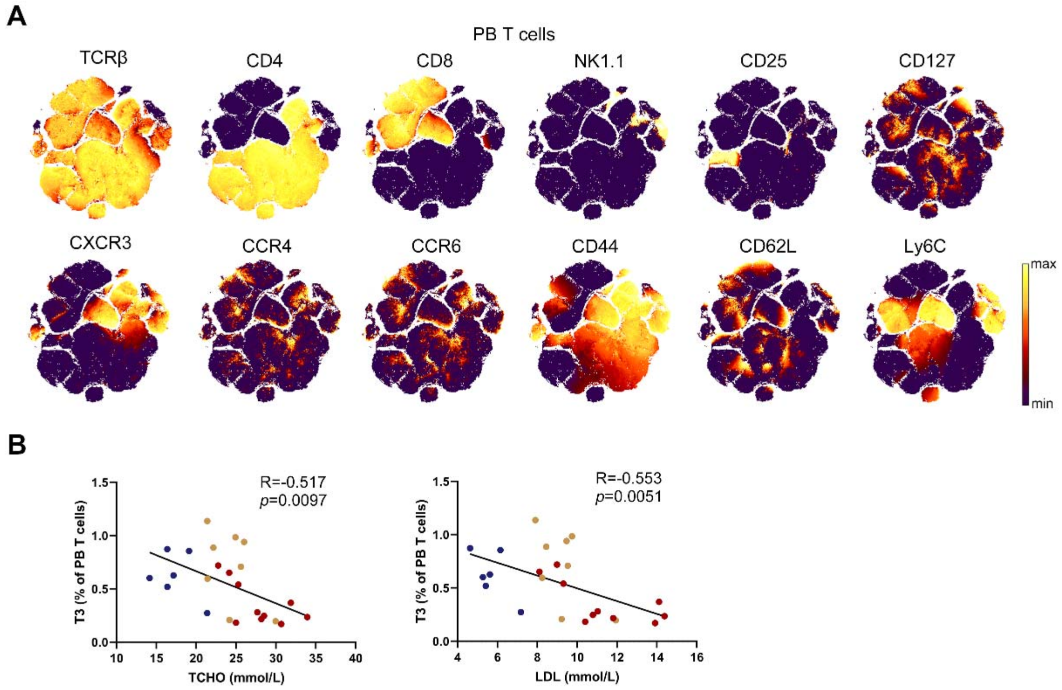

Figure S5. HO-1 inducer and inhibitor changed multiple T cells in the PB. (A) viSNE map showing the distribution of the total PB T cells from three groups. Cells on the viSNE map were colored by the normalized expression of indicated surface markers. (B) Dot plots (n=24) showing the Pearson correlation coefficients for relationships between the concentrations of total (TCHO) or LDL cholesterol in peripheral blood and the frequency of T3 in the PB T cells. Correlations were determined by a Pearson test. Only correlationships with |R|>0.4 and p<0.05 are shown.

Supplementary Table

Table S1. Mass cytometry antibody reagents

**Label**	**Target**	**Ab clone**	**Dilution**	**Source**
89Y	CD45	30-F11	1:100	Fluidigm
141Pr	Ly6G	1A8	1:100	Fluidigm
142Nd	CD11c	N418	1:100	Fluidigm
143Nd	TCRβ	H57-597	1:100	Fluidigm
145Nd	CD4	RM4-5	1:100	Fluidigm
146Nd	F4/80	BM8	1:100	Fluidigm
148Nd	CD11b (Mac-1)	M1/70	1:100	Fluidigm
149Sm	CD19	6D5	1:100	Fluidigm
150Nd	Ly6C	HK1.4	1:100	Fluidigm
151Eu	CD25 (IL-2R)	3C7	1:100	Fluidigm
152Sm	CD3e	145-2C11	1:100	Fluidigm
156Gd	CD196 (CCR6)	29-2L17	1:100	Fluidigm
160Gd	CD62L (L-selectin)	MEL-14	1:100	Fluidigm
162Dy	CD44	IM7	1:100	Fluidigm
165Ho	CD161 (NK1.1)	PK136	1:100	Fluidigm
168Er	CD8a	53-6.7	1:100	Fluidigm
169Tm	CD206 (MMR)	C068C2	1:100	Fluidigm
170Er	CD49b	HMa2	1:100	Fluidigm
171Yb	CD80 (B7-1)	16-10A1	1:100	Fluidigm
172Yb	CD86	GL1	1:100	Fluidigm
174Yb	CD127 (IL-7Ra)	A7R34	1:100	Fluidigm
176Yb	CD45R (B220)	RA3-6B2	1:100	Fluidigm
163Dy	CD183(CXCR3)	CXCR3-173	1:100	BioLegend
175Lu	CCR4	2G12	1:100	BioLegend
159Tb	CD103	2E7	1:100	BioLegend
114Cd	IA/IE(MHC II)	M5/114.15.2	1:100	BioLegend
